# Family-Based Telehealth Initiative to Improve Nutrition and Physical Activity for Children With Obesity and Its Utility During COVID-19: A Mixed Methods Evaluation

**DOI:** 10.3389/fnut.2022.932514

**Published:** 2022-07-11

**Authors:** Melissa N. Poulsen, Jennifer Franceschelli Hosterman, G. Craig Wood, Adam Cook, Lyndell Wright, Scott T. Jamieson, Allison Naylor, Shawnee Lutcher, Jacob Mowery, Christopher J. Seiler, Gregory J. Welk, Lisa Bailey-Davis

**Affiliations:** ^1^Department of Population Health Sciences, Geisinger, Danville, PA, United States; ^2^Center for Nutrition and Weight Management, Geisinger, Danville, PA, United States; ^3^Obesity Institute, Geisinger, Danville, PA, United States; ^4^Department of Kinesiology, Iowa State University, Ames, IA, United States

**Keywords:** COVID-19, family-based intervention, nutrition, pediatric obesity, physical activity, rural

## Abstract

Guidelines recommend primary care providers refer children with obesity to behavioral interventions, but given limited program availability, access, and parental engagement, referrals remain rare. We developed telehealth coaching interventions for families whose children received care at a health system in Pennsylvania, United States in 2019-2020. Intervention referrals were facilitated by the pediatrician and/or project team for 6–12-year-old children with obesity following well-child visits. Participants chose one of three 26-week interventions focused on healthy eating, physical activity, or a hybrid clinical/nutrition intervention. Interventions engaged parents as change agents, enhancing self-efficacy to model and reinforce behavior and providing resources to help create a healthy home environment. We enrolled 77 of 183 eligible parent/child dyads. We used mixed methods to evaluate the interventions. Repeated measures models among participants showed significant reductions in obesogenic nutrition behaviors post-intervention and at 1-year follow-up, including a reduction in sugar-sweetened beverage intake of 2.14 servings/week (95% confidence interval: −3.45, −0.82). There were also improvements in obesoprotective nutrition behaviors (e.g., frequency of family meals, parental self-efficacy related to meal management). One year post-baseline, we observed no significant differences in changes in body mass index (BMI) z-scores comparing child participants with matched controls. Given potential impacts of COVID-19 community restrictions on study outcomes, we conducted qualitative interviews with 13 participants during restrictions, which exemplified how disrupted routines constrained children’s healthy behaviors but that intervention participation prepared parents by providing cooking and physical activities at home. Findings support the potential of a telehealth-delivered nutrition intervention to support adoption of healthy weight behaviors.

## Introduction

Childhood obesity is a pervasive public health challenge that demands innovative solutions. Nearly one in five children in the United States has obesity (1) and almost 6% have severe obesity, with children in rural areas among the most affected (2). Children with obesity have increased risk for chronic disease early in life (3), and if left untreated, obesity often persists into adulthood (4). Interventions often target risk and protective factors based on a biopsychosocial model, replacing unhealthy behaviors with new ones (5), but few have been implemented at scale to benefit high risk populations (1, 6).

Family-based interventions that help establish and maintain healthy lifestyle behaviors such as healthy eating and physical activity comprise an essential approach for preventing and managing obesity, particularly for preadolescents (5, 7). According to Social Cognitive Theory (SCT) (8), primary caregivers shape the home environment, which is a key external context that influences child behavior. Furthermore, caregivers play a primary role in children’s observational learning by modeling, encouraging, and reinforcing health behaviors (9, 10).

Pediatric primary care provides a key setting for obesity risk screening and initiating family-based interventions, with opportunity for scalable benefit since most US children routinely attend well-child visits (11). The US Preventive Services Task Force (USPSTF) recommends providers screen for obesity in children 6 years and older and offer those with obesity comprehensive behavioral intervention with at least 26 contact hours over a 2–12-month period (12). Offering interventions involves referrals to outside programs, but US pediatricians report a lack of referral resources (13). Accordingly, intervention referral rates remain strikingly low, despite high need (14). Families in the US and elsewhere also face barriers in completing referrals, including time constraints, competing demands, travel distance, and childcare needs (15–18), which render USPSTF recommendations largely unattainable. Telehealth provides a promising approach to intervention delivery that can mitigate access barriers.

This paper describes Enhanced PREVENT, a set of family-based telehealth interventions for 6–12-year-old children with obesity. We report participation outcomes, changes in children’s healthy eating and physical activity and nutrition-related family practices following intervention participation, and post-intervention changes in child body mass index *z*-scores (BMIz) as compared to a set of control children. Notably, the COVID-19 pandemic and related societal restrictions emerged during the project’s intervention phase. Given the potential impact of these restrictions—which included school closures, leaving children homebound and likely at higher risk for obesity (19, 20) — on study outcomes, we report interview findings from participants regarding children’s health behaviors during COVID-19 restrictions, challenges faced in supporting such behaviors, and the interventions’ utility in preparing caregivers to mitigate these challenges.

## Methods

### Study Design and Setting

A quality improvement initiative was designed for rural school-aged children with overweight or obesity attending a well-child visit and their primary caregiver (hereafter, “parent”). The interventions were delivered and evaluated in Geisinger’s service region, a rural area of Pennsylvania, from April 2019 to May 2020. Pediatrician counseling on obesity prevention is supported through institutionalized screening called PREVENT (21). As standard of care at well-child visits, parents complete the Family Nutrition and Physical Activity (FNPA) tool (22) and results are integrated into children’s electronic health record (EHR). Results identify modifiable, obesogenic factors and provide pediatricians an opportunity to discuss patient-centered prevention strategies. The current initiative aimed to identify models that enhance secondary prevention (Enhanced PREVENT).

We evaluated the initiative using a mixed methods approach with a concurrent embedded design (23). Post-intervention changes in children’s eating and physical activity and nutrition-related family practices were evaluated among participants with a pre-post design. Change in BMIz was evaluated using a case-matched controlled design with a non-equivalent control group. Less of a priority, the embedded qualitative component allowed exploration of the models’ value as families adapted to emergent COVID-19 related restrictions. We conducted semi-structured telephone interviews with parent participants in May and June 2020, 9–13 weeks following statewide restrictions due to COVID-19. Pennsylvania’s initial pandemic response involved school closures in mid-March 2020 alongside statewide stay-at-home orders on April 1st. Phased, partial re-opening of businesses within the project area began in May and June. The Geisinger Institutional Review Board determined the initiative to be non-research.

### Interventions

**FIGURE 1:**
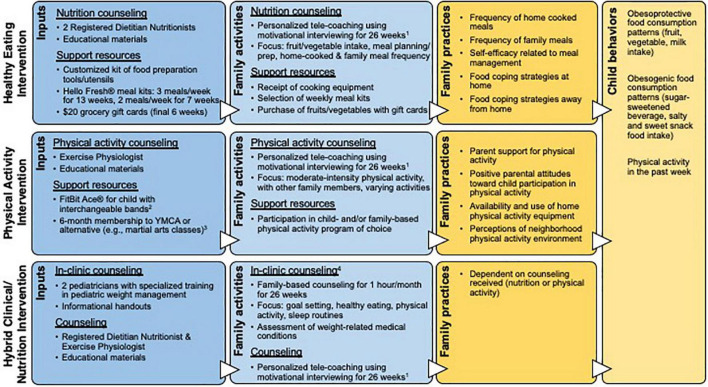
Logic models for the three interventions, depicting intervention inputs, outputs (family activities), short-term outcomes (family practices), and medium-term outcomes (child behaviors). ^1^Healthy eating and physical activity intervention counseling components included a 1-h initial session, 30-min sessions weekly over weeks 2–13 and biweekly over weeks 14–20, and monthly 15-min calls in weeks 21–26. The hybrid intervention included one telehealth coaching session per month over 26 weeks. ^2^FitBit^®^ provided to motivate child participants to engage in and monitor their physical activity and as a counseling tool; data were not used as a behavioral outcome. ^3^The YMCA membership was modified from individual to family membership in September 2019 to increase enrollment. ^4^Clinical costs were covered by Geisinger Health Plan, which includes a medical assistance option.

We developed three family-based interventions with input from a patient advisory council on obesity and pediatricians to target families’ concerns and encourage healthy lifestyle behaviors: healthy eating, physical activity, and hybrid clinical/nutrition (Figure 1). Families were given intervention choice to involve them in decision-making and optimize participation. Each intervention leveraged healthcare system and community resources with the intent of developing care delivery solutions that enhance prevention. Interventions were family-based in that parent/child dyads were asked to participate in sessions and interventionists recommended family-based practices.

The healthy eating and physical activity interventions included approximately 600 min of individualized telehealth coaching over 26 weeks and aimed to change child behavior through parental influence and modification of the home environment. Interventions were informed by SCT, incorporating the social influence parents have on children through behavioral modeling (e.g., eating meals or exercising together), self-efficacy in skill development (e.g., meal preparation, setting screentime limits), establishment of healthy routines (e.g., regular family meals or physical activity), and behavioral reinforcement (e.g., internal knowledge of optimal food/activity choices) (8–10). Other SCT components included goal setting and environmental supports. During coaching sessions, parents participated in problem solving and set goals, and were accountable for discussing family progress at subsequent sessions. The interventions supported parents in modifying the home environment through procurement of food or physical activity resources. Healthy eating participants received healthy meal kit home-delivery, grocery gift cards, and cooking equipment [need based on an inventory (24)]. Physical activity participants received a YMCA membership and FitBit ^®^ for the child. Pertinent to this paper, the healthy eating intervention’s cost was estimated to be $2,348 US dollars (2020) per family per year (including registered dietitian/nutritionist hourly rate with benefits, average meal kit cost, 75% participation in telehealth sessions and meal kit deliveries, and the average cost of cooking utensils).

The hybrid clinical/nutrition intervention represented adaptation of the current standard of care, as the program has been available for in-person delivery at Geisinger since 2008, but reach was historically low. We modified the intervention by delivering the nutrition component *via* telehealth coaching (hence it was ‘hybrid’ clinical and telehealth). To minimize modification of the existing program and ensure it could be sustained by the health system, families were not provided support resources.

### Participants and Recruitment

Participants included parent/child dyads. Sample size was restricted by available funding. Inclusion criteria were applied to children: aged 6–12.9 years, age- and sex-specific BMI ≥ 85th percentile, and attended a well-child visit at one of three participating pediatric clinics during the project period. Exclusion criteria included: child diagnosed with a psychiatric disorder, food allergies, dietary restrictions, or physical impairments limiting physical activity that would interfere with participation; family did not have internet access.

Participants were recruited through referral by their pediatrician during a well-child visit or by the project team following the visit. The project team identified potential child participants based on EHR eligibility criteria. Ten days after sending an invitation letter, the project team contacted parents by phone to explain the project and screen for additional exclusion criteria. Parents completed a consent form online and selected their intervention.

To compare child BMIz change, we used the EHR to retrospectively select a comparison group of child controls who were eligible for the project but who did not participate. The control cohort was derived from children who completed well-child visits at one of the three clinics during the recruitment period. For each of the 62 child participants with a 1-year post-baseline BMI measure, three controls were randomly selected without replacement from the pool of controls that met matching criteria. Matching criteria included age, sex, and baseline BMI. The resulting set of 186 matched controls had a similar distribution of age, sex, race, ethnicity, and BMI (Supplementary Table 1).

### Measures

The FLEX 7-day Food Frequency Questionnaire measured consumption patterns over the previous week (25). The questionnaire estimates weekly servings and quantities of multiple food items within five food categories (beverages, fruits, vegetables, salty snacks, sweet snacks) and evaluates the number of days respondents ate out (including takeout) in the last week. We assessed the frequency of home cooked meals (26) and meals eaten as a family (27) in the past week. Given the emphasis of the intervention on food preparation and meal planning, we assessed self-efficacy related to meal management and food coping strategies at home and away-from-home (28). The Physical Activity Questionnaire for Older Children (29) assessed children’s physical activity in the last week. Designed for use during in-school periods, we modified the questions to also apply to non-school periods.

We also evaluated change in BMIz, adjusted for child age and sex (30). Child BMI was derived from weight and height measures taken during well-child visits, which were obtained from the EHR at baseline and 1-year follow-up. Given the downturn in pediatric visits during the pandemic (31) that limited 1-year follow-up BMI measures, families were provided home kits to assess and self-report children’s weight and height (32) to supplement missing EHR data. Sex-specific BMI-for-age percentiles identified children’s weight status: overweight (≥85th to <95th), obese (≥95th), and severely obese (≥120% of 95th).

Child age, sex, race/ethnicity, parent age and education, household income, household food security (33), participation in a government food program and FNPA (if incomplete at well-child visit) were collected during phone recruitment.

Interview questions focused on parents’ perceptions of children’s health behaviors during COVID-19 restrictions, challenges faced in helping children engage in healthy behaviors, and intervention utility in preparing parents to mitigate these challenges.

### Procedures

We assessed nutrition and physical activity behaviors and family practices *via* online questionnaires using REDCap electronic data capture (34, 35) at baseline, 1-month post-intervention (7-months after baseline), and 1-year after baseline. Participants received $20 gift cards for each questionnaire. Children participated in completing behavior-related questionnaire items in an age-dependent manner.

Interviewees were purposively selected to represent each intervention. Interviews lasted approximately 30 min and were audio-recorded and transcribed verbatim. Participants provided verbal consent and received a $40 gift card.

### Data Analysis

Statistical analyses were conducted using SAS version 9.4 (SAS Institute, Cary, NC, United States). Baseline characteristics of participants were compared by intervention groups using Fisher’s exact test for categorical data and analysis of variance for continuous data. Small sample sizes limited power to evaluate participants separately by intervention, thus primary results combine all participants. In supplemental analyses, we provide results separately by intervention for healthy eating and physical activity; the hybrid intervention’s sample size was too small to allow for separate analysis.

Associated changes in behaviors and practices comparing baseline to the two post-intervention follow-up periods were evaluated using repeated measures models (SAS PROC GLIMMIX). Linear models were used to evaluate continuous variables and multinomial models for ordinal variables. Effect sizes are reported as beta coefficients or odds ratios and 95% confidence intervals (CI), respectively.

Differences in BMIz changes from baseline to 1-year follow-up were compared between study participants and matched controls in two ways. First, we compared change in the proportion of children in three clinically meaningful groups (12) using a Cochran-Armitage trend test. The groups included: increase in BMIz of ≥0.25, stable BMIz (change in BMIz between −0.25 and +0.25) and decrease in BMIz of ≥0.25. Second, we compared mean change in BMIz using difference-in-differences repeated measures regression. We also compared child BMIz change between healthy eating participants that had at least 600 min of telehealth coaching (the full planned intervention time) vs. dyads that received less than 600 min. We compared BMIz groups as described above and compared mean BMIz change using a *t*-test. We excluded participants in the hybrid clinical/nutrition intervention, which included less planned telehealth coaching time, and the physical activity intervention, as there was only one dyad that met the 600 min threshold.

We employed a modified framework approach (36) to the analysis of interview transcripts. Following familiarization with the data, we develop a codebook based on the interview guide (deductive) and emergent themes (inductive). We coded transcripts using HyperResearch 4.5.0 (ResearchWare Inc., 2012). We then developed code summaries, integrating findings across interviewees, and mapped codes onto key themes.

## Results

The project team identified 321 children as eligible through the EHR. Of those, 187 were screened by telephone, resulting in 183 (98%) eligible; 73 (40%) enrolled in the project. Four children enrolled after pediatrician referral, resulting in 77 enrolled dyads. Four participants withdrew and seven were lost to follow-up after completing baseline questionnaires.

Participant selection of interventions included 40 in healthy eating (filled first and capped due to budget restrictions), 31 in physical activity, and six in hybrid. Reasons parents gave for selecting the healthy eating intervention included expectations that child involvement in food selection and/or meal preparation could improve the child’s diet and wanting assistance with healthy cooking, healthier food choices, or meal planning. Some selected this intervention because the physical activity intervention was not feasible or because their child was already physically active. Likewise, some parents chose the physical activity intervention because nutrition was already an area of focus for their family. Most parents chose the physical activity intervention because they believed their child should be more active. Parents reported selecting the hybrid intervention because it was perceived to cover multiple behaviors.

Among the 77 enrolled dyads, children were primarily White non-Hispanic (Table 1). At enrollment, 48% were overweight, 32% were obese, and 19% were severely obese. Of the 66 participants who started an intervention, 53% completed ≥75% of the intervention sessions. At this completion threshold, participation was highest for hybrid (100% of dyads), followed by healthy eating (68% of dyads) and physical activity (29% of dyads) interventions. On average, dyads completed 77% of healthy eating sessions and 50% of physical activity sessions. The median (interquartile range) time for total telehealth coaching time was 575 (398, 695), 165 (30, 225), and 165 (137, 184) min for the health eating, physical activity, and hybrid interventions, respectively.

**TABLE 1:** Characteristics of parent/child dyads enrolled in Enhanced PREVENT, by intervention.

Characteristic	Healthy eating (n = 40)	Physical activity (n = 31)	Hybrid clinical/ Nutrition (n = 6)	*P*-value^1^
Child age in years, mean (SD)	9.4 (2.0)	9.6 (1.7)	8.4 (1.7)	0.354
Child female sex, n (%)	20 (50%)	13 (42%)	1 (17%)	0.342
Child BMI z-score, mean (SD)	1.7 (0.4)	1.8 (0.5)	2.0 (0.5)	0.311
Child BMI percentile, n (%)				0.151
>85th–94th	22 (55%)	13 (42%)	2 (33%)	
≥95th	14 (35%)	10 (32%)	1 (17%)	
≥120% 95th	4 (10%)	8 (26%)	3 (50%)	
Child race/ethnicity, n (%)				0.606
White, non-Hispanic	37 (93%)	27 (87%)	5 (83%)	
Black, non-Hispanic	3 (8%)	4 (13%)	1 (17%)	
Parent age in years, mean (SD)	39.6 (7.7)	36.3 (8.3)	45.2 (9.3)	0.035
Parent highest level of education, n (%)				0.196
<12 years	0 (0%)	2 (6%)	1 (17%)	
High school	7 (18%)	11 (35%)	1 (17%)	
College	20 (50%)	12 (39%)	2 (33%)	
Graduate school	9 (23%)	3 (10%)	1 (17%)	
Other	4 (10%)	3 (10%)	1 (17%)	
Household income, n (%)				0.434
<$25 k	6 (15%)	10 (32%)	1 (17%)	
$25–49 k	8 (20%)	6 (19%)	1 (17%)	
$50–99 k	12 (30%)	5 (16%)	2 (33%)	
≥$100 k	8 (20%)	3 (10%)	2 (33%)	
Unknown	6 (15%)	7 (23%)	0 (0%)	
Household food security, n (%)				0.934
Secure	28 (70%)	22 (71%)	5 (83%)	
Low	5 (13%)	4 (13%)	0 (0%)	
Very low	3 (7%)	1 (3%)	0 (0%)	
Unknown	4 (10%)	4 (13%)	1 (17%)	
Government food				0.014
program participation^2^, n (%)	7 (18%)	15 (48%)	1 (17%)	
Family gym membership, n (%)				0.567
Yes	9 (17%)	3 (10%)	0 (0%)	
No	29 (73%)	23 (74%)	5 (83%)	
Unknown	4 (10%)	5 (16%)	1 (17%)	
Family pool membership, n (%)				0.343
Yes	11 (28%)	7 (23%)	3 (50%)	
No	25 (62%)	20 (64%)	2 (33%)	
Unknown	4 (10%)	4 (13%)	1 (17%)	
FNPA score^4^, mean (SD)	60.8 (7.1)	60.3 (7.31)	60.8 (5.2)	0.954

*BMI, body mass index; FNPA, family nutrition and physical activity; SD, standard deviation. ^1^P-values from ANOVA for continuous variables or Fisher’s exact test for categorical variables.^2^WIC, SNAP, free or reduced-price National School Lunch Program. ^3^Scores range from 20 to 80 (sum of items with scores of 1 = never/almost never to 4 = almost always/always); lower scores indicate a higher obesogenic environment.*

Post-intervention questionnaires were completed by 47 (70%) dyads at 7-months and 52 (78%) dyads at 1-year post baseline. All nutrition-related behaviors and practices trended in the hypothesized direction following intervention (Table 2). Average sugar-sweetened beverage intake decreased by 2.14 (95% CI: −3.45, −0.82; *p*-value: 0.002) servings per week from baseline to post-intervention follow-up. Similarly, average intake of sweet snack foods decreased by 2.80 (95% CI: −4.75, −0.84; *p*-value: 0.006) servings per week. We also observed statistically significant changes for self-efficacy related to meal management and most food coping strategies at home and away-from-home. Comparison of effects by intervention choice showed larger average improvements in these nutrition-related behaviors and practices in families receiving the healthy eating intervention compared to those in the physical activity intervention (Supplementary Table 2). There was no evidence of a change in child physical activity behavior (Table 2).

**TABLE 2:** Associated changes (beta coefficients for linear models; odds ratios for multinomial models) in child behaviors and family practices comparing baseline to post-intervention and 12-month follow-up among study participants^1^.

Measure	Post-intervention follow-up		One-year follow-up	
	Estimate (95% CI)	*P*-value	Estimate (95% CI)	*P*-value
**Nutrition-related behaviors (intake)**				
Fruit	β = 2.27 (0.19, 4.35)	0.033	β = 1.42 (−0.64, 3.48)	0.173
Vegetables	β = 1.75 (−0.25, 3.76)	0.085	β = 1.98 (−0.01, 3.96)	0.050
Milk	β = 0.10 (−0.95, 1.16)	0.844	β = −0.85 (−1.90, 0.20)	0.111
Sugar-sweetened beverages	β = −1.95 (−3.27, −0.63)	0.004	β = −2.14 (−3.45, −0.82)	0.002
Salty snack foods	β = −1.54 (−3.16, 0.08)	0.063	β = −1.40 (−3.00, 0.20)	0.085
Sweet snack foods	β = −2.43 (−4.40, −0.45)	0.017	β = −2.80 (−4.75, −0.84)	0.006
**Obesoprotective nutrition-related practices**				
Home cooked meal frequency^2^	OR = 3.16 (1.31, 7.66)	0.011	OR = 2.97 (1.21, 7.30)	0.018
Family meal frequency^2^	OR = 3.33 (1.27, 8.71)	0.015	OR = 2.69 (1.03, 7.03)	0.044
**Self-efficacy related to meal management^3^**				
Meal planning	OR = 5.07 (2.11, 12.15)	<0.001	OR = 2.60 (1.13, 5.92)	0.024
Choosing healthy food at store	OR = 6.64 (2.67, 16.50)	<0.001	OR = 3.63 (1.53, 8.62)	0.004
Cooking for the family	OR = 4.11 (1.65, 10.20)	0.003	OR = 3.18 (1.29, 7.86)	0.013
**Food coping strategies at home^4^**				
Determine weekly menu	OR = 2.93 (1.22, 7.03)	0.017	OR = 1.93 (0.78, 4.73)	0.151
Make weekly grocery list	OR = 3.86 (1.64, 9.05)	0.002	OR = 2.35 (1.00, 5.52)	0.049
Cooking with few ingredients	OR = 2.99 (1.29, 6.93)	0.011	OR = 3.66 (1.51, 8.88)	0.005
Prepare meals in advance	OR = 3.62 (1.55, 8.39)	0.003	OR = 2.89 (1.22, 6.82)	0.016
Double recipes	OR = 2.64 (1.15, 6.04)	0.022	OR = 2.46 (1.06, 5.73)	0.037
**Food coping strategies away-from-home**				
Frequency of eating out^5^	OR = 0.22 (0.08, 0.57)	0.002	OR = 0.19 (0.07, 0.50)	0.001
Eating in a sit-down restaurant^6^	OR = 0.19 (0.08, 0.45)	<0.001	OR = 0.04 (0.01, 0.11)	<0.001
Eating in a fast-food restaurant^6^	OR = 0.28 (0.12, 0.66)	0.004	OR = 0.13 (0.05, 0.35)	<0.001
Using delivery and takeout^6^	OR = 0.76 (0.34, 1.69)	0.494	OR = 0.52 (0.23, 1.18)	0.115
Buying “ready to eat” foods^6^	OR = 0.17 (0.07, 0.44)	<0.001	OR = 0.14 (0.05, 0.38)	<0.001
**Physical activity behavior**				
Physical activity score^7^	β = −0.10 (−0.32, 0.12)	0.382	β = 0.08 (−0.15, 0.30)	0.493

*CI, confidence interval; OR, odds ratio. ^1^Sample sizes vary due to missing data. ^2^Weekly frequency ranged from 0 to 7. ^3^Responses ranged from 1 (low self-efficacy) to 8 (high self-efficacy). ^4^Responses ranged from 1 (non-alignment with strategy) to 5 (high alignment). ^5^Daily frequency ranged from 1to 6. ^6^Frequency of meals in past month ranged from 1 (never) to 5 (very often). ^7^Physical activity in past 7 days; scores ranged from 1 (low) to 5 (high).*

We obtained a BMI measurement at 1-year follow up for 62 (89%) of child participants; four of these were self-reported measures. Although changes in BMIz from baseline to 1-year follow-up were in the hypothesized direction for child participants as compared to matched controls, these differences were not statistically significant (Table 3). Outcomes appeared slightly more beneficial for healthy eating, vs. physical activity, intervention participants in terms of mean change in BMIz and the proportion of children with a decrease in BMIz (Supplementary Table 3). Healthy eating intervention participants with greater total telehealth coaching time similarly trended toward improved BMIz outcomes, but differences were not statistically significant (Table 3).

**TABLE 3:** Change in body mass index *z*-score from baseline to 1-year post-intervention follow-up, comparing **(A)** study participants with matched controls, and **(B)** healthy eating intervention participants dichotomized by total telehealth coaching time.

Group	N	% of individuals			Trend *P*-value^1^	Mean change in BMIz (SD)	*T*-test *P*-value^2^
		Increase in BMIz ≥ 0.25	Stable BMIz within 0.25	Decrease in BMIz ≤ −0.25			
**(A) Analysis of participants and matched controls**							
Study participants	62	19%	66%	15%	0.123	+0.03 (0.33)	0.111^2^
Matched controls	186	26%	65%	9%		+0.10 (0.28)	
**(B) Analysis by total telehealth coaching time among health eating intervention participants**							
<600 min	20	30%	55%	15%	0.204	+0.09 (0.46)	0.195^3^
≥600 min	16	13%	63%	25%		−0.10 (0.38)	

*BMIz, body mass index z-score; SD, standard deviation. ^1^Cochran–Armitage trend test. ^2^Difference-in-difference repeated measures model. ^3^T-test of mean change for each group.*

We interviewed 13 parent participants. Interviewees represented a mix of employment situations; many were newly working remotely. None described financial impacts such as job loss resulting from the pandemic nor food insecurity. Findings revealed a mix of obesogenic and obesoprotective behaviors among children arising during the shutdown (Supplementary Table 4). Changes in nutrition-related behaviors included increased snacking due to boredom and accessibility, reduced restaurant food consumption due to restaurant closures and being homebound, and increased home cooking, with some families reporting more family meals. Parents adapted grocery shopping practices to avoid virus exposure, including shopping less frequently, using curbside pickup, and not bringing children. All parents said the shutdown impacted children’s physical activity, whether through closure of facilities, cancelation of organized sport activities, not having physical education and recess, or not playing with other children. Most reported that children’s overall activity levels declined. Although parents compensated by encouraging outdoor play, several commented they were unable to make up for lost activity. For many children, screen time reportedly increased due to being home and bored. When asked how Enhanced PREVENT participation helped them manage during the shutdown, healthy eating participants reported feeling better prepared by being in the routine of cooking and having healthy recipes on hand. Physical activity participants reported having additional ideas available for encouraging activity at home (e.g., using a resistance band).

## Discussion

Through telehealth delivery of a family-based coaching intervention serving rural school-aged children with overweight or obesity, Enhanced PREVENT removed barriers to intervention access. Intervention choice allowed families to focus on behaviors of concern, potentially motivating participation. We observed improvements in healthy lifestyle behaviors and family practices post-intervention, particularly for the healthy eating intervention, and non-significant trends suggesting a potential benefit of participation on child BMI, particularly with greater telehealth coaching time. The interventions also may have helped families support children’s healthy behaviors during the COVID-19 pandemic.

We utilized system-level and family-centered implementation strategies previously shown to be effective (37) to increase enrollment. The FNPA tool supported family-centered weight discussions and intervention referral during pediatric well-child visits. Yet recruitment occurred mainly through the project team. Time limitations and lack of patient-facing materials may have constrained provider referrals. Family-centered strategies included telehealth delivery of coaching sessions, designed to reduce participation barriers identified in past studies (15), and intervention choice. Reasons provided by families for intervention selection centered on their preferred behavioral focus, suggesting that intervention choice allowed families to select the area of most concern, potentially motivating participation. The healthy eating intervention had the highest enrollment and participation, suggesting it met a need among families. The physical activity intervention had the lowest session participation and intervention “dose,” an important factor to intervention success (38). The hybrid clinical/nutrition intervention was least popular, with only six participants. Though not explicitly stated by participants, we hypothesize that the required in-person clinic visits presented a barrier. The hybrid intervention also lacked support resources that may have incentivized enrollment in the other interventions.

All post-intervention nutrition-related behavior and practice outcomes trended in the hypothesized direction. We observed significant and meaningful (39) changes in children’s food intake, including reduced consumption of sugar-sweetened beverages and sweet snack foods. We also observed significant changes for nearly all nutrition-related family practices. Larger effect sizes were generally observed in these nutrition-related measures among healthy eating vs. physical activity intervention families. In contrast, we found no evidence of changes in child physical activity. The relative success of the healthy eating intervention may be due to its attendance to internal, behavioral, and environmental facilitators, key features of the reciprocal interaction described by SCT (8). The intervention engaged parents, who indirectly involved children through meal selection, preparation, and shopping. This model engages parents as change agents and may enhance self-efficacy for obesoprotective practices such as providing home cooked meals, as demonstrated in a prior study (40). Nutrition coaching also focused on the home setting where eating behaviors are practiced. Support resources reinforced changes to the home eating environment, thereby ensuring parents had equipment and food to prepare healthy meals, factors that have previously been associated with family meal frequency and child consumption of family meals (24). In contrast, the physical activity intervention may have insufficiently influenced internal and external environments necessary to encourage greater child physical activity. For example, while telehealth coaching encouraged home physical activity, support resources provided did not alter the home environment where parents have the most influence.

Multi-component interventions with 26 contact hours have consistently been associated with clinically meaningful changes in BMIz; multi-component interventions with 6–25 contact hours have had inconsistent results (41). To reduce participation barriers, our interventions were remote (or hybrid) with ten planned telehealth coaching hours over the 26-week intervention period. We observed no significant change in child BMIz comparing participants with controls, but observed a trend toward greater decrease in BMIz among intervention children. Our results are similar to those reported in a review of multi-component interventions with 6–25 contact hours (41). More promising evidence emerged when we looked at “dose” of telehealth coaching time. Healthy eating intervention participants who received the full planned intervention time of 600 min experienced a BMIz decrease greater than those with less contact hours, and this decrease was greater than that observed with multi-component interventions (41). When combined with the observed improvements in nutrition-related behavior and practice outcomes described above, the nutrition intervention appears promising. However, our findings suggest that additional telehealth coaching time may be necessary to produce an effect on BMI. Importantly, the results of this study may be diluted by effects of the COVID-19 pandemic, as children experienced significant increases in the rate of BMI change during the pandemic, particularly among children with overweight, obesity, and severe obesity (42).

Telehealth delivery was fortuitous as intervention delivery was not compromised during the emergence of the COVID-19 pandemic and its associated community restrictions. Interview findings suggest that intervention participation prepared parents to maintain obesoprotective behaviors and practices during COVID-19 restrictions by pre-establishing healthy habits and providing new ideas about cooking and physical activities at home. Additionally, being homebound with children created opportunities to utilize intervention strategies such as having children help with cooking, which can have lasting benefits on diet quality (43). However, other intervention strategies were interrupted by the pandemic. For example, avoiding bringing children grocery shopping hampered parents’ ability to involve children in selecting healthy foods, a strategy encouraged during telehealth coaching sessions to enhance children’s interest in trying new foods (44). Additionally, the physical activity intervention’s focus on involving children in organized activities likely limited its impact during the shutdown.

This project was subject to several limitations. As a small quality improvement initiative, it was designed to identify models to enhance preventative care, not to compare intervention effectiveness. Recruitment was limited to central Pennsylvania; further study is needed to test the generalizability of findings to other rural settings. With participants self-reporting behaviors, results may be subject to social desirability bias and the Hawthorne effect. We did not have full information on non-participating parents and so do not know whether there were systematic differences between participants and non-participants. Selection bias may therefore be a concern, particularly in the comparison of change in BMIz, as controls included individuals who declined participation. Although intervention choice was a key component of the project, we had to cap participation in the healthy eating intervention, leaving some families with only two intervention options from which to choose. The cost of the support resources may limit replication, potentially necessitating adaptation. A limited number of follow-up BMI measures were obtained through self-report, though a previously evaluated tool kit was utilized to increase accuracy (32). Finally, qualitative findings regarding COVID-19 restrictions may not be transferable to other populations, as interviewees were (coincidentally) all female and represented a rural, largely White population who did not report being financially impacted by the pandemic. Nonetheless, consistencies with prior studies highlight common themes regarding the shutdown’s adverse consequences on childhood obesity.

Despite limitations, Enhanced PREVENT yielded promising results, providing a model for family-centered implementation strategies, including telehealth delivery and intervention choice to enhance participation. Significant changes in outcomes and demand for the healthy eating intervention support this approach to improving families’ healthy eating practices, suggesting an opportunity for future evaluation in a larger trial. Further, the COVID-19 pandemic has shown the home environment to be essential to healthy lifestyle behaviors and the need for interventions to incorporate at-home strategies.

## Data Availability Statement

The datasets presented in this article are not readily available because of the need to protect patient confidentiality and participant privacy. Requests to access the datasets should be directed to LB-D, ldbaileydavis@geisinger.edu.

## Ethics Statement

The Geisinger Institutional Review Board determined the study to be non-research; thus, ethical approval was not required in accordance with local legislation and institutional requirements. Primary caregiver participants provided electronic consent prior to participating in the study.

## Author Contributions

MP, JH, LW, SJ, AN, GJW, and LB-D contributed to the conception and design of the study. AC identified the potential participants in the electronic health record. SL managed the recruitment and primary data collection. JM managed the distribution of intervention support tools. CS managed the intervention implementation. LW, SJ, and AN conducted the telehealth coaching. JH and LW conducted the hybrid intervention. GCW performed the statistical analysis. MP and LB-D wrote the first draft of the manuscript. All authors reviewed and approved the submitted version of the manuscript.

## Conflict of Interest

The authors declare that the research was conducted in the absence of any commercial or financial relationships that could be construed as a potential conflict of interest.

## Publisher’s Note

All claims expressed in this article are solely those of the authors and do not necessarily represent those of their affiliated organizations, or those of the publisher, the editors and the reviewers. Any product that may be evaluated in this article, or claim that may be made by its manufacturer, is not guaranteed or endorsed by the publisher.
