# Supplementary material for: Family-Based Telehealth Initiative to Improve Nutrition and Physical Activity for Children With Obesity and Its Utility During COVID-19: A Mixed Methods Evaluation
Source: Front Nutr. 2022 Jul 11;9:932514. doi: 10.3389/fnut.2022.932514 (PMC9309788; doi:10.3389/fnut.2022.932514)
Supplement: Supplementary file 1 [file Data_Sheet_1.pdf]

Supplementary Material

Supplemental Table 1. Characteristics of child intervention participants and matched controls[1]

**Characteristic**	**Intervention participants** (n = 62)	**Controls** (n = 186)	***p*-value**[2]
Child age in years, mean (SD)	9.2 (1.9)	9.5 (1.8)	0.266
Child female sex, n (%)	29 (47%)	87 (47%)	0.999
Child race/ethnicity, n (%)			0.469
White, non-Hispanic	59 (95%)	179 (96%)	
Black, non-Hispanic	3 (5%)	4 (2%)	
Other	0 (0%)	3 (2%)	
Child BMI z-score, mean (SD)	1.80 (0.44)	1.79 (0.44)	0.926
Child BMI percentile, n (%)			0.675
$>85^{th}$-94th	28 (45%)	77 (41%)	
$\geq 95^{th}$	22 (35%)	78 (42%)	
$\geq$120% 95th	12 (19%)	31 (17%)	

[1]Matching criteria included age (within 12 months), sex, and baseline BMI (for the majority of controls, within three percentiles and measured within two months of the baseline BMI of the corresponding case, otherwise within five percentiles). [2]*P*-values from two-sample t-test for continuous variables or Fisher's exact test for categorical variables.

Supplemental Table 2. Associated changes (beta coefficients for linear models; odds ratios for multinomial models) in child behaviors and family practices comparing baseline to post-intervention and one-year follow-up among study participants, by intervention arm[1]

Measure	Post-intervention follow-up		One-year follow-up	
	**Estimate (95% CI)**	**p-value**	**Estimate (95% CI)**	**p-value**
Nutrition-related behaviors (intake)				
Fruit				
Healthy eating intervention	β = 0.35 (-1.91, 2.60)	0.757	β = -0.03 (-2.14, 2.07)	0.974
Physical activity intervention	β = 4.86 (0.77, 8.94)	0.022	β = 3.40 (-0.78, 7.58)	0.107
Vegetables				
Healthy eating intervention	β = 1.08 (-1.40, 3.56)	0.383	β = 0.03 (-2.28, 2.34)	0.978
Physical activity intervention	β = 2.45 (-1.16, 6.05)	0.176	β = 4.84 (1.15, 8.53)	0.012
Milk				
Healthy eating intervention	β = 0.22 (-1.02, 1.47)	0.721	β = -1.56 (-2.71, -0.40)	0.010
Physical activity intervention	β = 0.67 (-1.03, 2.38)	0.428	β = 0.77 (-0.97, 2.51)	0.373
Sugar-sweetened beverages				
Healthy eating intervention	β = -2.28 (-3.95, -0.60)	0.009	β = -2.14 (-3.72, -0.56)	0.009
Physical activity intervention	β = -0.85 (-3.07, 1.38)	0.443	β = -1.54 (-3.79, 0.72)	0.174
Salty snack foods				
Healthy eating intervention	β = -2.65 (-5.12, -0.17)	0.037	β = -2.81 (-5.11, -0.50)	0.018
Physical activity intervention	β = 0.13 (-2.02, 2.28)	0.903	β = 1.00 (-1.20, 3.20)	0.360
Sweet snack foods				
Healthy eating intervention	β = -3.32 (-6.47, -0.18)	0.039	β = -3.67 (-6.64, -0.71)	0.017
Physical activity intervention	β = -0.74 (-3.33, 1.85)	0.564	β = -1.45 (-4.10, 1.19)	0.271
Obesoprotective nutrition-related practices				
Home cooked meal frequency[2]				
Healthy eating intervention	OR = 4.47 (1.24, 16.13)	0.023	OR = 4.29 (1.24, 14.86)	0.023
Physical activity intervention	OR = 3.95 (0.88, 17.84)	0.072	OR = 2.36 (0.49, 11.42)	0.275
Family meal frequency[2]				
Healthy eating intervention	OR = 3.01 (0.87, 10.50)	0.081	OR = 4.42 (1.25, 15.65)	0.023
Physical activity intervention	OR = 5.67 (0.82, 39.31)	0.077	OR = 1.24 (0.24, 6.56)	0.790
Self-efficacy related to meal management[3]				
Meal planning				
Healthy eating intervention	OR = 9.79 (2.61, 36.72)	0.001	OR = 4.32 (1.38, 13.49)	0.013
Physical activity intervention	OR = 2.18 (0.58, 8.12)	0.236	OR = 1.18 (0.29, 4.87)	0.811
Choosing healthy food at store				
Healthy eating intervention	OR = 40.71 (7.98, 207.7)	<0.001	OR = 9.41 (2.60, 34.05)	0.001
Physical activity intervention	OR = 1.81 (0.48, 6.89)	0.369	OR = 2.02 (0.46, 8.91)	0.338
Cooking for the family				
Healthy eating intervention	OR = 5.52 (1.55, 19.63)	0.010	OR = 3.50 (1.09, 11.24)	0.036
Physical activity intervention	OR = 1.77 (0.40, 7.73)	0.436	OR = 1.99 (0.40, 9.97)	0.389
Food coping strategies at home[4]				
Determine weekly menu				
Healthy eating intervention	OR = 3.47 (1.01, 11.94)	0.049	OR = 4.09 (1.18, 14.17)	0.028
Physical activity intervention	OR = 1.99 (0.45, 8.73)	0.349	OR = 0.24 (0.04, 1.40)	0.108
Make weekly grocery list				
Healthy eating intervention	OR = 5.95 (1.64, 21.52)	0.008	OR = 3.03 (0.93, 9.87)	0.065
Physical activity intervention	OR = 2.12 (0.59, 7.61)	0.239	OR = 1.52 (0.38, 6.15)	0.545

Cooking with few ingredients				
Healthy eating intervention	OR = 3.67 (1.12, 11.98)	0.032	OR = 9.15 (2.49, 33.63)	0.001
Physical activity intervention	OR = 4.21 (0.99, 18.02)	0.052	OR = 1.81 (0.42, 7.87)	0.416
Prepare meals in advance				
Healthy eating intervention	OR = 8.20 (2.50, 26.92)	<0.001	OR = 4.13 (1.33, 12.81)	0.015
Physical activity intervention	OR = 1.38 (0.34, 5.63)	0.645	OR = 0.86 (0.17, 4.28)	0.849
Double recipes				
Healthy eating intervention	OR = 6.41 (1.89, 21.72)	0.004	OR = 3.61 (1.15, 11.36)	0.029
Physical activity intervention	OR = 0.78 (0.20, 3.07)	0.710	OR = 1.24 (0.28, 5.46)	0.769
Food coping strategies away-from-home				
Frequency of eating out[5]				
Healthy eating intervention	OR = 0.20 (0.05, 0.77)	0.021	OR = 0.29 (0.09, 0.99)	0.048
Physical activity intervention	OR = 0.16 (0.03, 0.83)	0.030	OR = 0.10 (0.02, 0.67)	0.020
Eating in a sit-down restaurant[6]				
Healthy eating intervention	OR = 0.22 (0.07, 0.74)	0.016	OR = 0.04 (0.01, 0.19)	<0.001
Physical activity intervention	OR = 0.15 (0.04, 0.59)	0.008	OR = 0.02 (0.01, 0.15)	<0.001
Eating in a fast-food restaurant[6]				
Healthy eating intervention	OR = 0.34 (0.09, 1.29)	0.109	OR = 0.15 (0.03, 0.63)	0.011
Physical activity intervention	OR = 0.32 (0.09, 1.15)	0.079	OR = 0.09 (0.02, 0.46)	0.005
Using delivery and takeout[6]				
Healthy eating intervention	OR = 0.52 (0.17, 1.60)	0.245	OR = 0.51 (0.17, 1.55)	0.229
Physical activity intervention	OR = 1.32 (0.38, 4.64)	0.656	OR = 0.48 (0.11, 2.02)	0.302
Buying “ready to eat” foods[6]				
Healthy eating intervention	OR = 0.07 (0.02, 0.28)	<0.001	OR = 0.10 (0.03, 0.38)	0.001
Physical activity intervention	OR = 0.18 (0.02, 2.11)	0.164	OR = 0.02 (0.01, 0.87)	0.043
Physical activity behavior				
Physical activity score[7]				
Healthy eating intervention	β = -0.40 (-0.72, -0.09)	0.013	β = -0.15 (-0.45, 0.15)	0.325
Physical activity intervention	β = 0.25 (-0.06, 0.55)	0.106	β = 0.37 (0.04, 0.70)	0.028

Abbreviations: confidence interval (CI); odds ratio (OR). [1]Sample sizes vary due to missing data. [2]Weekly frequency ranged from 0-7. [3]Responses ranged from 1 (low self-efficacy) to 8 (high self-efficacy). [4]Responses ranged from 1 (non-alignment with strategy) to 5 (high alignment). [5]Daily frequency ranged from 1-6. [6]Frequency of meals in past month ranged from 1 (never) to 5 (very often). [7]Physical activity in past 7 days; scores ranged from 1 (low) to 5 (high).

Supplemental Table 3. Change in body mass index z-score from baseline to one-year post-intervention follow-up, comparing study participants in the (A) healthy eating and (B) physical activity interventions with matched controls

Group	N	% of individuals			Trend *p*-value[1]	Mean change in BMIz (SD)	*p*-value[2]
		Increase in BMIz ≥ 0.25	Stable BMIz within 0.25	Decrease in BMIz ≤ -0.25			
(A) Analysis of healthy eating intervention participants and matched controls							
Study participants	36	22%	58%	19%	0.293	+0.016 (0.393)	0.280
Matched controls	108	25%	65%	10%		+0.080 (0.286)	
(B) Analysis of physical activity intervention participants and matched controls							
Study participants	22	18%	73%	9%	0.221	+0.063 (0.249)	0.217
Matched controls	66	32%	62%	6%		+0.142 (0.261)	

Abbreviations: BMIz, body mass index z-score; SD, standard deviation. [1]Cochran-Armitage trend test. [2]Difference-in-difference repeated measures model.

Supplemental Table 4. Interview themes with illustrative quotations from parents who participated in Enhanced PREVENT

**Theme**	**Quotation**
Increased snacking	*The children want to eat more of what they want to eat because it is so easy to get to the refrigerator now, or snack cupboard ... because you are at home. ... Because children say they are bored…* – Parent of 10-year-old boy
Decreased eating out	*I think that's probably the biggest one, is the whole not running kids around, not trying to grab any fast food. ... I just think that prior to [the pandemic], if we felt hurried, out of convenience, we may grab something on the run. It may not necessarily be fast food. It may have been from a little restaurant nearby, but it's much quicker food and eating out, and that's not happening now.* – Parent of 8-year-old girl
Cooking at home and more frequent family meals	*I was totally against getting takeout through almost all the quarantine until the last two weeks we got our very first takeout. So, we've been cooking suppers every night.* – Parent of 9-year-old girl<br>*I think we have more time to plan things and/or make things as a family more. I think it's all good positive things. … [W]e all are eating pretty much every meal together. ...because things are closed down, [the kids are] just staying closer to home, which is great.* – Parent of 13-year-old boy
Altered grocery shopping practices and children’s involvement in shopping	*The kids don't shop with me anymore. I've only been doing on-line orders… So usually they help[ed] me. We always [went] to farmer's market and we haven't done that. ... I don't want them exposed to anything.* – Parent of 10-year-old boy<br>*We were getting into having [child] pick new things at the store just to try, things that are better and healthier, which I haven't taken him to a store at all since the beginning of March.* – Parent of 11-year-old boy
Altered grocery purchases and higher bills	*You can't find chicken now and we're even struggling to find meats, so I would say we have bought more fish, but we have also tried things that we never tried before like prepared meatballs. I usually make those myself... so more prepared type foods ... that we normally haven't [purchased] that aren't as ‘clean’...* – Parent of 10-year-old boy<br>*The produce section—there's not as much there because of supply chain. When we were [in Enhanced PREVENT], we were trying new fruits and vegetables, and we have kind of backed off of that because it's not available.* – Parent of 10-year-old boy

	I have noticed my grocery bill has almost doubled and we're okay right now with that, but if it keeps going up, I could see how that would create more of a time to buy cheaper food which isn't as good for you. – Parent of 10-year-old boy
Children's involvement in cooking	*I think the food part of [the restrictions] has been a lot better and also just with my kids having to cook or learning to cook more and actually really good cooks.* – Parent of 13-year-old boy<br>*[S]he enjoys baking, so one of the things we do since she is at home more is she tends to bake more. It's hard, we've have had more treats available just because that's an activity that she enjoys, but we try to look at healthier options for recipes.* – Parent of 9-year-old girl
Fewer opportunities for physical activity	*[T]hat was probably the worst, the organized activities just flat out shutting down. …she has been very deep into soccer and having nonstop sporting activities and track was coming up, and she was on swim team, and then all of a sudden, it's just gone. … I couldn't even make up for that, even if the weather was good… I'm not one to be able to just run around the yard for hours, trying to keep my kids highly engaged.* – Parent of 8-year-old girl<br>*It's also hard because [he is] not able to go and do things with friends or see friends. That is when [child] would go and just run around with kids literally in the yard or whatever.* – Parent of 13-year-old boy
Outdoor activity	*[T]hey have really greatly reduced his schooling. So, because of that, he is outside a whole lot more. He gets up in the morning and just wanting to be outside. …other than work, we aren't doing any other activities or going anywhere, so we're actually able to be outside with him more and encourage him to be outside more.* – Parent of 8-year-old boy<br>*We live in the middle of central Pennsylvania, so there are lots of places to walk, so we've been walking several miles a day, and [child] has been right there beside me the whole time.* – Parent of 9-year-old girl
Increased screen time	*There is a lot of time where she is bored and when the weather was bad, we weren't as strict as we sometimes can be about it. …she definitely has more TV time, because there is a lot of time where I was working… there is a lot of time to fill that I can't be completely present there... We did not have a lot of screen time prior, with all the activities and school and work. Now, there is more opportunity for it.* – Parent of 8-year-old girl
Altered sleep schedule	*I would say she isn't sleeping as well because she's going to bed later but she's still waking up pretty early and then I just kind of feel like her schedule is off. She seems a little bit more restless. She is actually taking naps a couple times here and there during the day, which is not typical*

	behavior for her … it has messed with it a little bit, [but] not to a concerning level. – Parent of 6-year-old girl
Parental coping with the shutdown	*I think this has made our life more difficult really. … It's a lot more work to be home, juggling kids and work, trying to work. … I can't do what another parent can do right now, like take their kid for bike ride when I have to go check my e-mail and deal with things. … I'm fighting with kids to do homework.* – Parent of 8-year-old girl<br>*I mean we have been fortunate to adjust pretty easily. And I work part-time and I can work from home and so we haven't been too impacted by childcare and I have a daughter that is old enough that could watch [child] if needed.* – Parent of 9-year-old girl
